# Safety and immunogenicity of a parenteral P2-VP8-P[8] subunit rotavirus vaccine in toddlers and infants in South Africa: a randomised, double-blind, placebo-controlled trial

**DOI:** 10.1016/S1473-3099(17)30242-6

**Published:** 2017-05-05

**Authors:** Michelle J Groome, Anthonet Koen, Alan Fix, Nicola Page, Lisa Jose, Shabir A Madhi, Monica McNeal, Len Dally, Iksung Cho, Maureen Power, Jorge Flores, Stanley Cryz

**Affiliations:** 1 South African Medical Research Council, Respiratory and Meningeal Pathogens Research Unit and Department of Science and Technology/National Research Foundation, Vaccine Preventable Diseases, University of the Witwatersrand, Johannesburg, South Africa; 2 PATH, Seattle, WA, USA; 3 National Institute for Communicable Diseases, National Health Laboratory Service, Sandringham, South Africa; 4 Department of Medical Virology, Faculty of Health Sciences, University of Pretoria, Pretoria, South Africa; 5 Division of Infectious Diseases, Cincinnati Children’s Hospital Medical Center, Cincinnati, Ohio, USA; 6 The Emmes Corporation, Rockville, MD, USA

## Abstract

**Background:**

Efficacy of live oral rotavirus vaccines is reduced in low-income compared with high-income settings. Parenteral non-replicating rotavirus vaccines might offer benefits over oral vaccines. We assessed the safety and immunogenicity of the P2-VP8-P[8] subunit rotavirus vaccine at different doses in South African toddlers and infants.

**Methods:**

This double-blind, randomised, placebo-controlled, dose-escalation trial was done at a single research unit based at a hospital in South Africa in healthy HIV-uninfected toddlers (aged 2 to <3 years) and term infants (aged 6 to <8 weeks, without previous rotavirus vaccination). Block randomisation (computer-generated, electronic allocation) was used to assign eligible toddlers (in a 6:1 ratio) and infants (in a 3:1 ratio) in each dose cohort (10 μg, followed by 30 μg, then 60 μg if doses tolerated) to parenteral P2-VP8-P[8] subunit rotavirus or placebo injection. The two highest tolerated doses were then assessed in an expanded cohort (in a 1:1:1 ratio). Parents of participants and clinical, data, and laboratory staff were masked to treatment assignment. P2-VP8-P[8] vaccine versus placebo was assessed first in toddlers (single injection) and then in infants (three injections 4 weeks apart). The primary safety endpoints were local and systemic reactions within 7 days after each injection, adverse events within 28 days after each injection, and all serious adverse events, assessed in toddlers and infants who received at least one dose. In infants receiving all study injections, primary immunogenicity endpoints were anti-P2-VP8-P[8] IgA and IgG and neutralising antibody seroresponses and geometric mean titres 4 weeks after the third injection. This trial is registered at ClinicalTrials.gov, number NCT02109484.

**Findings:**

Between March 17, 2014, and Sept 29, 2014, 42 toddlers (36 to vaccine and six to placebo) and 48 infants (36 to vaccine and 12 to placebo) were enrolled in the dose-escalation phase, in which the 30 μg and 60 μg doses where found to be the highest tolerated doses. A further 114 infants were enrolled in the expanded cohort between Nov 3, 2014, and March 20, 2015, and all 162 infants (12 assigned to 10 μg, 50 to 30 μg, 50 to 60 μg, and 50 to placebo) were included in the safety analysis. Serum IgA seroresponses were observed in 38 (81%, 95% CI 67–91) of 47 infants in the 30 μg group and 32 (68%, 53–81) of 47 in the 60 μg group, compared with nine (20%, 10–35) of 45 in the placebo group; adjusted IgG seroresponses were seen in 46 (98%, 89–100) of 47 infants in the 30 μg group and 47 (100%; 92–100) of 47 in the 60 μg group, compared with four (9%, 2·5–21) of 45 in the placebo group; and adjusted neutralising antibody seroresponses against the homologous Wa-strain were seen in 40 (85%, 72–94) of 47 infants in both the 30 μg and 60 μg groups, compared with three (7%, 1·4–18) of 45 participants in the placebo group. Solicited reactions following any injection occurred with similar frequency and severity in participants receiving vaccine and those receiving placebo. Unsolicited adverse events were mostly mild and occurred at a similar frequency between groups. Eight serious adverse events (one with placebo, two with 30 μg, and five with 60 μg) occurred in seven infants within 28 days of any study injection, none of which were deemed related to study treatment.

**Interpretation:**

The parenteral P2-VP8-P[8] vaccine was well tolerated and immunogenic in infants, providing a novel approach to vaccination against rotavirus disease. On the basis of these results, a phase 1/2 trial of a trivalent P2-VP8 (P[4], P[6], and P[8]) subunit vaccine is underway at three sites in South Africa.

**Funding:**

Bill & Melinda Gates Foundation.

## Introduction

Rotavirus is a common cause of severe diarrhoea in children younger than 5 years, with the highest burden of disease observed in low-income countries in Africa and Asia.^1,2^ Live oral rotavirus vaccines have shown reduced efficacy in low-income countries compared with high-income countries.^3–7^ This observation is consistent with the diminished immunogenicity and effectiveness of other live oral vaccines, such as polio and cholera, in low-income countries.^8,9^ Interference by high titres of rotavirus antibodies acquired transplacentally, micronutrient deficiency, malnutrition, interfering gut microflora, enteric co-infections, concomitant disease, and differences in rotavirus epidemiology might contribute to this suboptimal performance.^10^

Research in context

**Evidence before this study**

We searched the PubMed database for trials published in English between Jan 1, 1983, and Oct 18, 2016, with the terms “rotavirus”, “vaccines”, and “trial”. Two oral rotavirus vaccines, Rotarix (monovalent human-derived vaccine; GlaxoSmithKline Biologicals, Rixensart, Belgium) and RotaTeq (pentavalent bovine-derived vaccine; Merck Vaccines, Whitehouse Station, NJ, USA) have been licensed and are recommended for global use in children by WHO. Clinical trials of these vaccines done in middle-income and high-income countries in Latin America, Europe, and USA have showed very good protective efficacy (85–98%) against severe rotavirus disease. However, lower efficacy (49–72%) and immunogenicity have been observed in clinical studies of rotavirus vaccines in low-income and middle-income countries in Africa. These efficacy levels are in keeping with the reduced performance observed in low-income countries of other live oral vaccines such as those targeting poliomyelitis, typhoid, and cholera, as well as previous rotavirus vaccine candidates. Differences in the epidemiology of rotavirus, micronutrient deficiency (zinc, vitamin A), malnutrition, interfering microbiota present in the gut, enteric viral and bacterial co-infections, and concomitant disease in infants, such as diarrhoea, tuberculosis, malaria, or HIV infection, in addition to coadministration with oral polio vaccine, might contribute to suboptimal immune responses in infants in low-income settings. Withholding breastfeeding at the time of rotavirus vaccination was not shown to improve immune responses to the oral rotavirus vaccines. However, high levels of maternal rotavirus antibodies might inhibit responses, and alternative dosing schedules such as a birth dose of rotavirus vaccine have been explored in view of the potential inhibition by maternal rotavirus antibodies with an early vaccine dose. Some of the gut-related issues associated with the live-attenuated oral vaccines might be circumvented by the development of parenterally administered non-replicating rotavirus vaccines, which could lead to improved efficacy. Additionally, these vaccines might have fewer safety risks (risk of intussusception), be less expensive to produce, and could be added to existing vaccines, thereby facilitating acceptability and delivery. Parenteral vaccines have been successfully used for other diseases caused by enteric pathogens—eg, poliovirus and cholera. Animal studies have shown that parenteral rotavirus vaccines, including inactivated virus, virus-like particles, and truncated VP8 subunit proteins can induce high serum rotavirus-specific antibody titres and neutralising activity. Several new rotavirus vaccine candidates are in various phases of development. A neonatal vaccine candidate RV3-BB was found to be immunogenic and well tolerated in phase 1 and 2 trials. A phase 1 trial done in the USA showed that a monovalent P2-VP8-P[8] subunit vaccine was well tolerated and immunogenic when administered to adults intramuscularly.

**Added value of this study**

This is the first study to our knowledge to administer a parenteral non-replicating rotavirus vaccine to an infant population. The study found that the monovalent P2-VP8-P[8] subunit rotavirus vaccine was well tolerated and immunogenic in young infants when administered parenterally at age 6, 10, and 14 weeks. Additionally, a reduction in the shedding of Rotarix, administered 1 month after the third P2-VP8-P[8] injection, suggests that the vaccine might be having an effect at the mucosal interface. This exploratory model could potentially be used for assessing other vaccines. Based on these study results, a phase 1/2 trial of a trivalent P2-VP8 (P[4], P[6], and P[8]) subunit vaccine began in early 2016 at three sites in South Africa.

**Implications of all the available evidence**

Providing a parenteral rotavirus vaccine has the potential to address safety concerns and the suboptimal efficacy of the oral rotavirus vaccines at a reduced cost in low-income and middle-income countries, which have a high burden of rotavirus disease. The monovalent P2-VP8-P[8] subunit rotavirus vaccine was well tolerated in this study and the previous phase 1 trial, showed good immunogenicity to the vaccine antigen, and provides a novel approach to vaccination against rotavirus disease using a parenteral route. Demonstration of reduced shedding of the Rotarix vaccine strain following P2-VP8-P[8] vaccination in this study raises the possibility of further exploring this model as a surrogate marker of field efficacy.

Parenterally administered, non-replicating rotavirus vaccines (NRRVs) bypass the need for intestinal replication of live oral vaccines, so might have enhanced efficacy.^11^ Both oral and parenteral vaccines have been successfully developed for other enteric diseases, such as polio, typhoid, and cholera. Post-marketing surveillance after the introduction of rotavirus vaccines has detected a small increase in the risk of intussusception following oral rotavirus vaccine administration, which could be avoided with NRRVs.^12^ Additionally, subunit protein vaccines can be produced at a very low cost and can potentially be combined with other childhood vaccines, thereby facilitating delivery and acceptability within routine immunisation programmes.

Two structural proteins in the outer capsid of the rotavirus, VP7 and VP4, have been characterised. The VP4 protein must be cleaved by proteases before the virus can be activated, resulting in the formation of two proteins, VP5 and VP8. Preclinical studies have shown that various NRRVs can induce serum rotavirus-specific binding and neutralising antibodies that are protective in experimental models.^13–16^ In particular, truncated VP8 subunit proteins have been shown to elicit high titres of homotypic neutralising antibodies and variable titres of heterotypic neutralising antibodies against different rotavirus strains in animal models.^15,16^ In a first-in-human clinical trial,^17^ a P2-VP8-P[8] subunit vaccine was found to be well tolerated and showed promising immunogenicity when administered intramuscularly to adults. This vaccine consists of a truncated VP8 subunit (aminoacids 64–223 of the protein) from the Wa strain (G1P[8]) of human rotaviruses fused with the P2 epitope from tetanus toxin and expressed in *Escherichia coli*.^15^ In this study, we assessed the safety and immunogenicity of the P2-VP8-P[8] subunit vaccine at different doses in South African toddlers and infants. We also assessed the effect of vaccination on shedding of the Rotarix vaccine virus strain.

## Methods

### Study design and participants

This single-centre, double-blind, randomised, placebo-controlled, dose-escalation trial assessed participants at the Respiratory and Meningeal Pathogens Research Unit based at the Chris Hani Baragwanath Academic Hospital (Johannesburg, South Africa), which serves the urban population of Soweto. The protocol was approved by the Human Research Ethics Committee, University of the Witwatersrand (Johannesburg, South Africa), the Western Institutional Review Board (Puyallup, WA, USA), and the Medicines Control Council (Pretoria, South Africa). We also submitted a US Food and Drug Administration Investigational New Drug Application, which was approved. The study was undertaken in accordance with South African Good Clinical Practice Guidelines.^18^

The dose-escalation phase was designed to test three dose levels (10 μg, 30 μg, and 60 μg) of vaccine, first in toddlers and then in infants. Cohorts of 14 toddlers (12 vaccine, two placebo) per dose level were to receive a single injection. Cohorts of 16 infants (12 vaccine, four placebo) per dose level were to receive three injections at 4-week intervals. The first participants were toddlers at 10 μg dose, followed by toddlers at 30 μg and infants at 10 μg dose, then toddlers at 60 μg and infants at 30 μg dose, and then infants at 60 μg dose. Progression from one dose to the next and from toddlers to infants required review by a safety review committee (SRC) of safety data for 7 days after the first injection in the respective dose or age cohort. The two highest tolerated dose levels were then assessed in an expanded cohort of 114 infants (38 at each dose level and 38 placebo).

Toddlers and infants were identified from hospital birth registers and postnatal wards, and invited for screening 1–7 days before randomisation. Healthy HIV-uninfected toddlers (aged ≥2 and <3 years) and infants (aged ≥6 and <8 weeks, ≥37 weeks gestation, and without previous receipt of rotavirus vaccination) were eligible for enrolment. Eligibility criteria were assessed through medical history, physical examination, and screening laboratory tests. Exclusion criteria included acute illness at time of enrolment, presence of malnutrition or any systemic disorder, congenital defects, known or suspected impaired immunological function, immunoglobulin therapy or chronic immunosuppressant medications, and concurrent participation in another clinical trial. Full inclusion and exclusion criteria are listed in the appendix p 1. Participants were only enrolled if their parents were literate and provided written informed consent, and intended to stay in the area with the child during the study.

### Randomisation and masking

Toddlers were randomly assigned to receive vaccine or placebo in groups of 14, beginning with 10 μg, then followed by 30 μg, then 60 μg. Within each group, randomisation was done in two blocks of seven toddlers (six were randomised to vaccine and one to placebo), in a random order within the block. Infants were randomly assigned in groups of 16 beginning with 10 μg, then followed by 30 μg, then 60 μg. Within each group, randomisation was done in four blocks of four infants (three to vaccine and one to placebo), in a random order within the block. Infants in the expanded cohort were allocated either to one of the two highest doses tolerated in the dose escalation phase, or to placebo, in blocks of three or six infants, with block size chosen at random and infants randomly ordered within the block in a 1:1:1 ratio. The randomisation sequence was computer generated by the Emmes Corporation (Rockville, MD, USA). A study investigator enrolled and randomly assigned participants electronically, and was provided with a blinded treatment number. This number was given to the unmasked pharmacist who prepared and dispensed the injection on the basis of the treatment number, in a masked syringe (taped to conceal the colour of the liquid), which was then administered intramuscularly into the thigh by a masked study investigator. Parents of participants and clinical, data, and laboratory staff were all masked to treatment assignment.

### Procedures

Toddlers in the dose-escalation phase of the trial received a single intramuscular injection of vaccine or placebo in the anterolateral thigh on the day of randomisation (day 0). Infants in both the dose-escalation phase and the expanded cohort received three intramuscular injections of vaccine or placebo in the anterolateral thigh on the day of randomisation (day 0) at age 6–7 weeks, day 28 at age 10–13 weeks, and day 56 at age 14–17 weeks (appendix p 6).

The P2-VP8-P[8] protein was produced at the Walter Reed Army Institute of Research, Pilot Bioproduction Facility (Silver Spring, MD, USA).^17^ The protein was diluted using sterile saline and formulated with aluminium hydroxide (Alhydrogel, Brenntag Biosector, Frederikssund, Denmark) by the study pharmacist within 6 h of administration to yield dose concentrations of 10 μg, 30 μg, and 60 μg per 0·5 mL containing 0·56 mg of aluminium. An injection of sterile saline was used as placebo. Vaccine or placebo in infants was given in the same thigh as hepatitis B vaccine (Heberbiovac-HB; The Biovac Institute, Cape Town, South Africa), which is given routinely to infants at ages 6, 10, and 14 weeks, whereas 13-valent pneumococcal conjugate vaccine (Prevenar13; Pfizer Laboratories, New York, NY, USA), routinely given at ages 6 and 14 weeks only, and the combination vaccine for diphtheria, tetanus, pertussis, poliomyelitis, and *Haemophilus influenzae* type b (Pentaxim; Sanofi Pasteur, Paris, France), routinely given at ages 6, 10, and 14 weeks, were given in the opposite thigh. Infants also received three doses of the oral Rotarix rotavirus vaccine (GlaxoSmithKline, Rixensart, Belgium) as part of this study, at 4, 8, and 12 weeks after the third study injection.

Participants were observed for 30 min after administration of each injection. Parents were provided with, and trained to correctly use, a thermometer, a tool to assess the size of injection site redness and swelling, and a memory aid booklet to assess and record local (injection site pain or tenderness, redness, swelling, and itching) and systemic (fever, vomiting, decreased appetite, irritability, and decreased activity) symptoms daily for 7 days after each injection. Clinic visits were done 3 and 7 days after each injection. Haemoglobin, white blood cell count with differential, platelet count, total bilirubin, albumin, creatinine, and alanine transaminase were assessed at baseline and 7 days after the first injection. Unsolicited adverse events were recorded from randomisation until the final study visit, 6 months after the last injection. Parents were advised to contact study staff if the child developed an adverse event during the course of the study. Grading and causality of adverse events were determined by the investigator using a grading scale developed for this study, and all safety data, including all adverse events, were reviewed by the SRC throughout the study. Study stopping rules are described in the appendix p 1.

Serum was collected at baseline, and at 4 weeks (all participants) and 24 weeks (infants only) after the final study injection (appendix p 6). Anti-P2-VP[8] IgG and IgA were quantified using standard ELISA assay techniques.^17^ Neutralising antibodies to Wa (G1P[8]), 89-12 (G1P[8]), DS-1 (G2P[4]), and 1076 (G2P[6]) were established as previously described.^19^ Rotavirus serum IgA was measured by an ELISA assay using whole virus lysate.^20^ Testing was done at the Division of Infectious Diseases, Cincinnati Children’s Hospital Medical Center (Cincinnati, OH, USA). Stool samples were collected from infants at 5, 7, and 9 days after the first dose of Rotarix and tested for the presence of rotavirus using the commercially available ProsPecT Rotavirus Microplate Assay (Oxoid Ltd, Ely, UK), according to the manufacturer’s instructions. ELISA-positive specimens were confirmed and genotyped by PCR amplification of the genes encoding VP7 and VP4 (appendix p 2). Testing was done at the National Institute for Communicable Diseases (Johannesburg, South Africa).

### Outcomes

The primary objectives were to assess the safety and reactogenicity of the P2-VP8-P[8] vaccine at escalating doses in toddlers and infants, and to investigate the immunogenicity at different doses in infants.

The primary safety endpoints were the number of serious adverse events, the number of adverse events within 28 days after each injection, and the number of vaccine-induced local and systemic reactions within 7 days after each injection and overall for the three combined injections. The primary immunogenicity endpoints were the proportion of infants with anti-P2-VP8 IgG and IgA seroresponses, the proportion of infants with neutralising antibody responses against rotavirus, anti-VP8 IgG and IgA geometric mean titres (GMTs) 4 weeks after the third injection in infants, and neutralising antibody GMTs 4 weeks after the third injection in infants. An unadjusted seroresponse was defined as an increase of four times or more in titre between baseline and 4 weeks after the third injection. Adjusted IgG and neutralising antibody post-injection titres accounted for the decay in maternal antibodies using the half-life calculated from participants in the placebo group who had detectable baseline titres that were higher than at the post-injection visit. This adjustment value was established for each assay separately. An adjusted seroresponse was defined as an increase of four times or more in titre between baseline and 4 weeks after the third injection (adjusted titre) in infants with an unadjusted post-injection titre greater than the limit of detection (the latter part of this definition was not included in the original statistical analysis plan but was added at the analysis stage). Neutralising antibody seroresponses were those against the strain from which the vaccine was based (homologous Wa strain) as well as against divergent rotavirus strains 89-12, DS-1, and 1079.

The secondary objective was to assess the effect of P2-VP8-P[8] vaccination on shedding of Rotarix vaccine virus subsequently administered in infants, with the endpoint being the proportion of infants shedding rotavirus (determined by ELISA) at 5, 7, or 9 days after administration of the first dose of Rotarix (4 weeks after the third P2-VP8-P[8] or placebo injection). Exploratory objectives were to assess the immunogenicity of one dose of P2-VP8-P[8] vaccine at different doses in toddlers, and to characterise the serum IgA response in infants receiving Rotarix after receiving the P2-VP8-P[8] vaccine. Endpoints were the proportion of toddlers with anti-P2-VP8 IgG and IgA seroresponses; the proportion of toddlers with neutralising antibody responses against rotavirus; anti-VP8 IgG and IgA GMTs and neutralising antibody GMTs 4 weeks after the single injection in toddlers; and anti-rotavirus IgA, anti-P2-VP8 IgG and IgA GMTs, and neutralising antibody GMTs in infants before and after Rotarix vaccination.

### Statistical analysis

The effect of the vaccine on the shedding of Rotarix vaccine virus was a secondary objective, but was the primary factor used to calculate the sample size for the dose escalation and expanded cohorts of infants. Establishing sample size in this manner means that the study has the statistical power to address both primary and secondary objectives, because the sample size needed for the secondary objective exceeded that required for the primary objectives. We calculated that 50 infants per dose group, allowing for 10% of participants to drop out, would enable detection of an 80% reduction in Rotarix virus shedding in recipients of the vaccine compared with placebo recipients (>80% power assuming ≥30% shedding in placebo recipients). This sample size provided a 90% or greater chance of observing an adverse event that had a 4·5% risk of occurrence, and more than 95% power to detect a 40 percentage point or greater difference in seroresponses between a vaccine group and the placebo group. Sample size calculations were based on Fisher’s exact test using a two-sided α of 0·05.

Toddlers and infants who received at least one dose of vaccine or placebo were assessed in the safety analysis. The unit of analysis was the proportion of participants with at least one event graded as moderate or worse (including solicited local reactions, systemic reactions, adverse events, and suspected adverse reactions), or any serious adverse events. The primary immunogenicity analysis included only infants who received all three study injections (per-protocol population). Logistic regression was used to detect differences in seroresponses between the treatment groups, including pair-wise comparisons if the overall difference was statistically significant. The Kruskal-Wallis test was used to compare magnitude of response between the treatment groups and Wilcoxon rank-sum test was used for pairwise comparisons. Assessment of shedding was done for each of the three specified post-Rotarix vaccination days, and for shedding on any of the 3 days combined. Infants for whom rotavirus was detected in any specimen by ELISA testing were considered to have undergone viral shedding, and the proportions of infants with shedding in the 30 μg and 60 μg dose groups and the combined 30 μg and 60 μg dose group were compared with the placebo group using logistic regression. The relative reduction in the proportion of participants with shedding of the Rotarix vaccine strain compared with the placebo group was analysed using the two-sample *t* distribution on log-transformed data.

Data were analysed using SAS software (version 9.3) and statistical significance defined as a two-tailed p<0·05. The trial was registered on ClinicalTrials.gov (NCT02109484).

### Role of the funding source

The funder of the study had no role in the study design, data collection, data analysis, data interpretation, or writing of the report. The corresponding author had full access to all the data in the study and had final responsibility for the decision to submit for publication.

## Results

**Figure 1:**
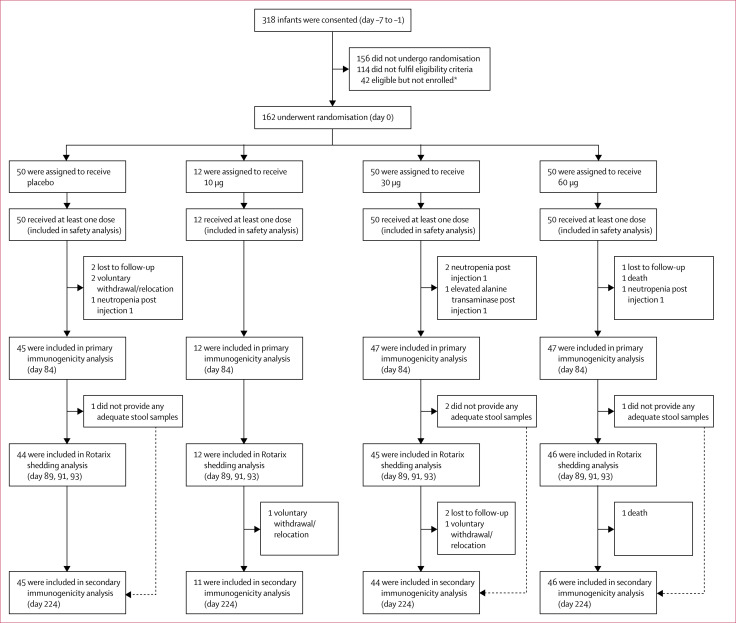
Trial profile for infants *16 enrolment postponed or enrolment into dosing group complete, 16 no longer interested, seven unsuccessful phlebotomy, three other.

From March 17, 2014, to Sept 29, 2014, 42 toddlers and 48 infants in the dose-escalation phase were eligible and randomly assigned to receive vaccine (36 toddlers and 36 infants) or placebo (six toddlers and 12 infants;). Because of an error during administration, one toddler who should have received placebo was given the 10 μg vaccine instead (appendix p 7). Figures and tables focus on results for infants; results for toddlers are in the appendix. The 30 μg and 60 μg doses were the two highest doses tolerated, so were used in the expanded infant group, for which a further 114 infants were randomly assigned to receive vaccine or placebo (figure 1). 151 infants received three injections of vaccine or placebo and had a serum sample collected 4 weeks after the final injection and were included in the primary immunogenicity analysis, 147 provided at least one stool sample after the first Rotarix injection so were included in the Rotarix shedding analysis, and 146 had a serum sample collected 24 weeks after the third injection, so were included in the secondary immunogenicity analysis (figure 1). Demographic characteristics were similar across treatment groups for toddlers and infants (table 1; appendix p 2).

**Table 1:** Baseline clinical and demographic characteristics in the infant treatment groups

	P2-VP8-P[8] vaccine 10 μg (n=12)	P2-VP8-P[8] vaccine 30 μg (n=50)	P2-VP8-P[8] vaccine 60 μg (n=50)	Placebo (n=50)
Age, days	46·2 (1·7)	46·5 (2·7)	47·1 (3·1)	46·2 (2·5)
Male sex	5 (42%)	23 (46%)	25 (50%)	24 (48%)
Black ethnicity	12 (100%)	49 (98%)^*^	50 (100%)	50 (100%)
Length, cm	54·5 (2·5)	54·7 (2·6)	55·7 (1·5)	55·3 (2·3)
Weight, kg	5·1 (0·7)	4·8 (0·6)	4·9 (0·5)	4·8 (0·6)

Data are n (%) or mean (SD).

* One infant (2%) had mixed ethnicity.

Solicited local and systemic reactions during the 7 days after the administration of any injection occurred with similar frequency and severity in the vaccine and placebo recipients. No severe or potentially life-threatening solicited adverse events were recorded in the toddlers, and most toddlers had mild or no solicited events (2 [5%] of 42 toddlers had moderate local reactions and 3 [7%] of 42 had moderate systemic reactions; appendix p 2). Most infants had mild or no solicited adverse events (11 [7%] of 162 infants had moderate or worse local reactions and 27 [17%] of 162 infants had moderate or worse systemic reactions; table 2; appendix p 3). Reports of events decreased in frequency as days passed after injection, and the distributions of reactions were similar between each post-injection period. Two infants had severe irritability, one in the 30 μg group (day 0, injection 2) and one in the placebo group (day 0, injection 3). The occurrence of these two events met prespecified study pause criteria. Review of individual case reports and cumulative infant reactogenicity data was done by the SRC, and they recommended that the study continue. One infant in the 60 μg group had a potentially life-threatening fever (axillary temperature 41·1°C) on day 1 after the third injection, which resolved without sequelae by day 2.

**Table 2:** Frequency of solicited (maximum reactogenicity per participant) and unsolicited (maximum severity per participant) adverse events in the infant treatment groups

	P2-VP8-P[8] vaccine 10 μg	P2-VP8-P[8] vaccine 30 μg	P2-VP8-P[8] vaccine 60 μg	Placebo
**Solicited local reactions during first 7 days after injection**				
After injection 1				
None or mild	12/12 (100%)	47/50 (94%)	48/50 (96%)	49/50 (98%)
Moderate or worse	0/12	3/50 (6%)	2/50 (4%)	1/50 (2%)
After injection 2				
None or mild	12/12 (100%)	47/47 (100%)	49/49 (100%)	44/47 (94%)
Moderate or worse	0/12	0/47	0/49	3/47 (6%)
After injection 3				
None or mild	12/12 (100%)	47/47 (100%)	47/48 (98%)	43/45 (96%)
Moderate or worse	0/12	0/47	1/48 (2%)	2/45 (4%)
Overall				
None or mild	12/12 (100%)	47/50 (94%)	47/50 (94%)	45/50 (90%)
Moderate or worse	0/12	3/50 (6%)	3/50 (6%)	5/50 (10%)
**Solicited systemic reactions during first 7 days after injection**				
After injection 1				
None or mild	12/12 (100%)	41/50 (82%)	48/50 (96%)	45/50 (90%)
Moderate or worse	0/12	9/50 (18%)	2/50 (4%)	5/50 (10%)
After injection 2				
None or mild	12/12 (100%)	45/47 (96%)	47/49 (96%)	42/47 (89%)
Moderate or worse	0/12	2/47 (4%)	2/49 (4%)	5/47 (11%)
After injection 3				
None or mild	12/12 (100%)	46/47 (98%)	47/48 (98%)	41/45 (91%)
Moderate or worse	0/12	1/47 (2%)	1/48 (2%)	4/45 (9%)
Overall				
None or mild	12/12 (100%)	40/50 (80%)	45/50 (90%)	38/50 (76%)
Moderate or worse	0/12	10/50 (20%)	5/50 (10%)	12/50 (24%)
Unsolicited adverse events during first 28 days after injection^*^				
After injection 1				
None or mild	12/12 (100%)	46/50 (92%)	47/50 (94%)	47/50 (94%)
Moderate or worse	0/12	4/50 (8%)	3/50 (6%)	3/50 (6%)
After injection 2				
None or mild	12/12 (100%)	46/47 (98%)	49/49 (100%)	47/47 (100%)
Moderate or worse	0/12	1/47 (2%)	0/49	0/47
After injection 3				
None or mild	12/12 (100%)	46/47 (98%)	48/48 (100%)	45/45 (100%)
Moderate or worse	0/12	1/47 (2%)	0/48	0/45
Overall				
None or mild	12/12 (100%)	44/50 (88%)	47/50 (94%)	47/50 (94%)
Moderate or worse	0/12	6/50 (12%)	3/50 (6%)	3/50 (6%)

Data are n/N (%).

* Serious adverse events are reported separately in the text and appendix p 4.

Unsolicited adverse events in toddlers were mostly mild (1 [2%] of 42 toddlers reported a moderate unsolicited adverse event), with no severe or serious adverse events reported (appendix p 2). Most infants had mild or no unsolicited, non-serious adverse events (12 [7%] of 162 infants had moderate or worse unsolicited events; table 2); the unsolicited events occurred at a similar frequency between treatment groups (table 2) and the most common were upper respiratory tract infection, nasal congestion, bronchiolitis, and cough. Five non-serious events graded as severe occurred within 28 days of any injection of vaccine or placebo. Two infants in the 30 μg dose-escalation group (one vaccine recipient, one placebo recipient) presented with the same severe laboratory abnormality (severe neutropenia^21^) after injection, which triggered a pause in study vaccination. No clinical adverse events were reported for either participant. Masked safety data, as well as unmasked white blood cell and differential counts, were reviewed by the SRC, which did not find support for an association with the study vaccine, and recommended that the study continue. Two infants in the expanded cohort (one 30 μg recipient and one 60 μg recipient) presented with severe neutropenia, and one infant (30 μg recipient) presented with severe increased alanine transaminase concentration. Infants with neutropenia and increased alanine transaminase concentration after the first injection did not receive further injections because of the temporal relationship between occurrence of the event and administration of the injection. These events were not assessed as serious and resolved spontaneously. Eight serious adverse events (one with placebo, two with 30 μg, and five with 60 μg) occurred in seven infants within 28 days of any study injection. None were assessed as having a reasonable possibility that the study product caused the event. One death occurred in a participant who received 60 μg; the infant had been admitted to hospital with severe pneumonia, was discharged, and died at home 13 days later, 1 day after receiving the third vaccination. After review of all available data by the SRC, the death was assessed as being unrelated to the study vaccine. 11 serious adverse events (three with placebo, five with 30 μg, and three with 60 μg) occurred more than 28 days after the third injection. These events included one death, 3·5 months after vaccination, for which the cause of death was not established. Descriptions of the serious adverse events are provided in the appendix p 4.

Unadjusted serum anti-P2-VP8-P[8] IgA seroresponse 4 weeks after the third injection was shown in seven (58%; 95% CI 28–85) of 12 infants in the 10 μg group, 38 (81%; 67–91) of 47 infants in the 30 μg group, and 32 (68%) of 47 (53–81) infants in the 60 μg group, compared with nine (20%; 10–35) of 45 in placebo recipients (table 3). Almost all vaccine recipients showed anti-P2-VP8-P[8] IgG seroresponses with adjusted rates of 98–100% in the vaccine groups (table 3). An adjusted neutralising antibody response to the Wa-strain was shown in 12 (100%) of 12 (74–100) infants in the 10 μg group, 40 (85%) of 47 (72–94) in the 30 μg and 60 μg groups, versus three (7%) of 45 (1·4–18) in the placebo group (table 3). Adjusted neutralising antibody responses to the 89-12 strain were also significantly different between the vaccine and placebo groups (table 3). Neutralising antibody responses to the DS-1 (P[4]) strain was higher in vaccine than placebo recipients, but no significant differences were detected in the 1076 (P[6]) strain between vaccine and placebo recipients. No significant differences were seen in any of the seroresponses between the 30 μg and 60 μg groups (data not shown).

**Table 3:** Serum antibody responses prevaccination and 4 weeks after the third injection of P2-VP8-P[8] vaccine or placebo in infants, according to treatment group

	Pre-vaccination GMT (95% CI)	After-vaccination GMT (95% CI)	Seroresponse, unadjusted		Seroresponse, adjusted^*^	
			n(%; 95% CI)	n(%; 95% CI)	p value^†^
**Anti-P2-VP8 IgA to P[8]**					
Placebo (n=45)	7 (5–8)	12 (8–17)	9 (20%; 10–35)	NA	NA
10 μg (n=12)	8 (3–18)	54 (16–182)	7 (58%; 28–85)	NA	0·0165
30 μg (n=47)	6 (5–7)	56 (39–82)	38 (81%; 67–91)	NA	<0·0001
60 μg (n=47)	6 (5–7)	38 (28–51)	32 (68%; 53–81)	NA	<0·0001
**Anti-P2-VP8 IgG to P[8]**					
Placebo (n=45)	95 (60–151)	32 (22–46)	1 (2%; 0·1–12)	4 (9%; 2·5–21)	NA
10 μg (n=12)	55 (23–128)	7155 (4372–11 709)	12 (100%; 74–100)	12 (100%; 74–100)	0·0006
30 μg (n=47)	107 (72–161)	9583 (7544–12 172)	46 (98%; 89–100)	46 (98%; 89–100)	<0·0001
60 μg (n=47)	166 (116–237)	9576 (8131–11 278)	46 (98%; 89–100)	47 (100%; 92–100)	<0·0001
**Neutralising antibodies to Wa strain (G1P[8])**					
Placebo (n=45)	76 (53–108)	13 (9–18)	0 (0–8)	3 (7%; 1·4–18)	NA
10 μg (n=12)	52 (23–115)	219 (107–449)	7 (58%; 28–85)	12 (100%; 74–100)	0·0004
30 μg (n=47)	86 (61–121)	212 (166–271)	17 (36%; 23–52)	40 (85%; 72–94)	<0·0001
60 μg (n=47)	123 (95–161)	195 (163–233)	7 (15%; 6–28)	40 (85%; 72–94)	<0·0001
**Neutralising antibodies to 89–12 strain (G1P[8])**					
Placebo (n=45)	95 (64–142)	20 (13–29)	1 (2%; 0·1–12)	4 (9%; 2·5–21)	NA
10 μg (n=12)	90 (40–205)	298 (147–604)	6 (50%; 21–79)	10 (83%; 52–98)	<0·001
30 μg (n=47)	105 (73–151)	400 (320–500)	24 (51%; 36–66)	42 (89%; 77–97)	<0·001
60 μg (n=47)	163 (122–218)	324 (250–419)	13 (28%; 16–43)	38 (81%; 67–91)	<0·001
**Neutralising antibodies to DS-1 strain (G2P[4])**					
Placebo(n=45)	60 (41–87)	11 (8–15)	0 (0–8)	4 (9%; 2·5–21)	NA
10 μg (n=12)	51 (22–119)	32 (11–94)	1 (8%; 0·2–39)	6 (50%; 21–79)	0·0028
30 μg (n=47)	71 (51–99)	26 (19–36)	3 (6%; 1·3–18)	15 (32%; 19–47)	0·0101
60 μg (n=47)	86 (63–117)	30 (23–39)	2 (4%; 0·5–15)	15 (32%; 19–47)	0·0101
**Neutralising antibodies to 1076 strain (G2P[6])**					
Placebo(n=45)	41 (30–56)	12 (9–16)	0(0–8)	4 (9%; 2·5–21)	0·2636
10 μg (n=12)	38 (15–96)	20 (7–56)	0(0–26)	2 (17%; 2·1–48)	··
30 μg (n=47)	46 (34–64)	18 (13–25)	2 (4%; 0·5–15)	11 (23%; 12–38)	··
60 μg (n=47)	67 (48–94)	26 (20–35)	1 (2%; 0·1–11)	11 (23%; 12–38)	··

GMT = geometric mean titre.

* IgG and neutralising antibody after-injection titres were adjusted for decay in maternal antibodies using the half-life calculated from participants in the placebo group who had detectable baseline titres that were higher than at the after-injection visit, and established for each assay separately. Half-life for IgG is 44·5 days; Wa is 32·1 days; 89–12 is 34·4 days; DS-1 is 30·2 days; 1076 is 41·0 days. Adjusted seroresponse was defined as a four-times or greater increase in titre between baseline and 4 weeks after the third injection (adjusted titre) in infants with an unadjusted post-injection titre greater than the limit of detection (16 for IgG; ten for neutralising antibodies).

† p value shows pair-wise comparison of each vaccine dose group with the placebo group, when the overall difference between dose groups was statistically significant, for IgA (unadjusted), IgG (adjusted), Wa (adjusted), and DS-1 (adjusted). For 1076, p value indicates overall treatment effect between groups (adjusted).

**Figure 2:**
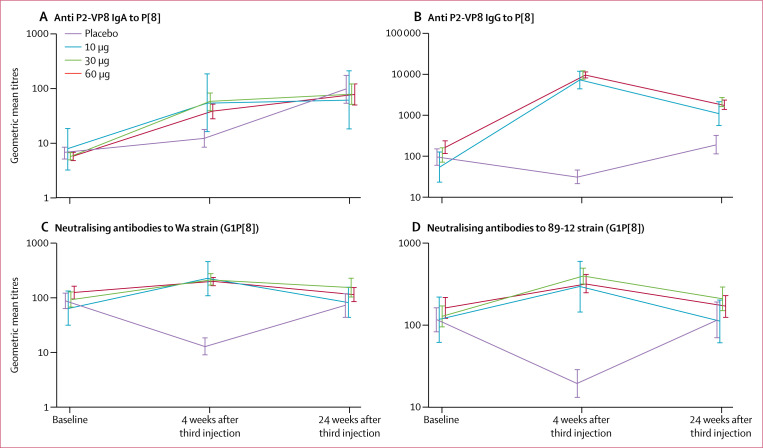
Serum antibody unadjusted geometric mean titres Measurements at baseline (day of screening), and 4 weeks (day 84) and 24 weeks (day 224) after the third injection of P2-VP8-P[8] vaccine or placebo. (A) Anti-P2-VP8-P[8] IgA; (B) anti-P2-VP8-P[8] IgG; (C) neutralising antibodies to Wa rotavirus strain; and (D) neutralising antibodies to 89-12 rotavirus strain. At 24 weeks, all infants had received three doses of Rotarix except one infant in the 10 μg group, three infants in the 30 μg group, and one infant in the 60 μg group, who received only one or two doses. Error bars show 95% CI.

24 weeks after the third injection of P2-VP8-P[8] vaccine or placebo (following three doses of Rotarix), serum anti-P2-VP8 IgG response was still significantly higher in the 30 μg (p=0·003) and 60 μg (p=0·002) groups than in the placebo group, whereas anti-P2-VP8 IgA and neutralising antibody responses to Wa and 89-12 strains were similar between the vaccine and placebo groups (figure 2). Rotavirus serum IgA GMTs to whole viral lysate were significantly higher 12 weeks after Rotarix administration than pre-Rotarix titres in all treatment groups, with no significant differences between groups (p=0·855; appendix p 5). Serum anti-P2-VP8 IgG and IgA GMTs before and after a single injection of vaccine or placebo in toddlers are shown in the appendix p 8.

At each timepoint after receipt of the first dose of Rotarix, the proportions of participants with shedding in the 30 μg and 60 μg groups were not significantly different to that in the placebo group (day 5 p=0·1683, day 7 p=0·1488, and day 9 p=0·6993). However, the proportion of participants with rotavirus shedding on at least one of the days was significantly lower in the 30 μg group (66% reduction, 95% CI 21–85; p=0·0087) and 60 μg group (49%, 0–75; p=0·0493) than in the placebo group (table 4). The difference between the 30 μg and 60 μg groups was not statistically significant on any of the days (p=0·4255). Rotavirus shedding was 57% (95% CI 23–76) lower in the combined group of 30 μg and 60 μg recipients than in placebo recipients (p=0·0052; table 4). The rotavirus strain was confirmed to be the Rotarix vaccine strain in 29 (91%) of 32 of the participants with viral shedding; the other three (one in 30 μg group and two in placebo) were a G9P[8] strain.

**Table 4:** Rotavirus shedding in the different treatment groups after administration of the first dose of Rotarix in infants receiving three injections of P2-VP8-P[8] vaccine or placebo

	Placebo	P2-VP8-P[8] vaccine 30 μg	P2-VP8-P[8] vaccine 60 μg	P2-VP8-P[8] vaccine 30 μg and 60 μg
**Day 5 after Rotarix**				
Number shedding	10/41 (24%)	3/38 (8%)	6/34 (18%)	9/72 (13%)
Reduction	NA	68% (0–90)	28% (0–71)	49% (0–77)
**Day 7 after Rotarix**				
Number shedding	11/40 (28%)	4/37 (11%)	6/40 (15%)	10/77 (13%)
Reduction*	NA	61% (0–86)	45% (0–78)	53% (0–78)
**Day 9 after Rotarix**				
Number shedding	5/36 (14%)	5/40 (13%)	3/38 (8%)	8/78 (10%)
Reduction*	NA	10% (0–72)	43% (0–85)	26% (0–74)
**Any timepoint**				
Number shedding	17/44 (39%)	6/45 (13%)	9/46 (20%)	15/91 (17%)
Reduction*	NA	66% (21–85)	49% (0–75)	57% (23–76)

Data are n/N (%) and % reduction (95% CI). The reduction is in comparison with the placebo group. NA=not applicable.

## Discussion

This study showed that the monovalent P2-VP8-P[8] subunit vaccine was well tolerated at 10, 30, and 60 μg without identifiable safety concerns. IgG responses to the vaccine antigen were high in magnitude, irrespective of whether the analysis included adjustment for maternal antibody titres. Although they were lower in magnitude than the IgG responses, most infants did show IgA responses. This study describes a novel approach to vaccinating infants against rotavirus disease using a non-replicating, parenterally administered P2-VP8-P[8] subunit vaccine. This approach could provide several advantages over the currently licensed live oral vaccines.

Despite numerous clinical trials investigating live oral rotavirus vaccine, no correlate of protection against rotavirus disease has been established. Studies suggest that serum IgA antibody detected by whole rotavirus lysate ELISA, which detects primarily anti-VP6 antibodies, might predict immunity conferred by live oral candidates, but the precise mechanism remains undefined.^22,23^ Undoubtedly, vaccination triggers additional, unidentified immune responses that contribute to oral rotavirus vaccine-induced protection. The correlate of protection for a protein-based rotavirus vaccine will probably be different, and we did not expect that the P2-VP8-P[8] vaccine would induce a robust antibody response to the whole viral lysate. Therefore, our measure of the immune response to vaccination included not only serum-binding antibodies to the vaccine antigen and neutralising antibodies to various strains of rotavirus, but also a novel functional assessment of the ability to suppress local gut multiplication of the vaccine strain contained in Rotarix, as established by a reduction in faecal shedding. Depending on the population, the proportion of infants receiving Rotarix who shed the vaccine strain varied after the first dose of Rotarix. However, few had viral shedding after receiving a second dose, which might be interpreted as evidence of a local protective immune response after the first dose.^24^ In this study, significantly fewer infants vaccinated with the 30 μg and 60 μg P2-VP8-P[8] vaccine shed rotavirus than did placebo recipients. These findings show that responses induced after vaccination with P2-VP8-P[8] could potentially be active at the gut surface in preventing virus multiplication. Whether the suppression of vaccine strain multiplication is mediated by homing of immune cells to the gut mucosa or by the leakage of serum antibody to the intestinal surface remains to be determined. Given the ethical challenge of undertaking placebo-controlled trials of new rotavirus vaccines, and the logistical and fiscal challenges of trials with an active comparator, identification of a correlate of protection and alternative methods are urgently needed to show efficacy of new rotavirus vaccines. Our findings raise the possibility of using this model to assess other rotavirus vaccines for their ability to induce a potentially protective immune response mediated at the gut surface.

Rotavirus strains expressing the P[8] type are responsible for the majority of rotavirus infections worldwide; however, P[4] and P[6] strains can account for up to 30% of isolates in Africa and southeast Asia.^25–27^ Although P[4], P[6], and P[8] antigens are extensively immunologically related, vaccination with the current P[8]-based vaccine elicited a strong neutralising antibody response against the two strains expressing the vaccine homologous P[8] type, but only a modest neutralising antibody response to heterologous P[4] strains, and no response to the P[6] strain. Although live oral rotavirus vaccines have been shown to provide protection against rotavirus gastroenteritis caused by rotavirus strains with and without G and P genotypes shared with the vaccine strain,^28,29^ this crossprotection might not occur with subunit vaccines, and a multivalent vaccine with P[4], P[6], and P[8] antigens might be required to provide protection against the common circulating rotavirus strains. Therefore, we are undertaking a multicentre study in South Africa to investigate the safety and immunogenicity of a trivalent P2-VP8-P[4/6/8] vaccine in adults, toddlers, and infants. Doses to be assessed in this trial range from 5 μg to 30 μg per serotype (15 μg to 90 μg total antigen) based on an absence of a clear dose–response relationship in the present study.

Our study had some limitations. Coadministration of the study vaccine with routine vaccines could have affected our ability to assess general safety and systemic reactogenicity of the study vaccine because some effects, especially systemic reactogenicity, could be due to the other vaccines. Although we could have offset study vaccines from the routine vaccines by 2 weeks, overlap of symptoms might still occur and, given the size of the study, we believed that the randomisation with the control group receiving placebo would allow for recognition of safety signals from the study vaccine. The safety results, with no meaningful or significant differences between vaccine and placebo recipients, appear to support the concomitant administration of the P2-VP8-P[8] vaccine and routine vaccinations. Although the majority of the infants shedding rotavirus were confirmed to be shedding the Rotarix vaccine strain, three infants shed a strain that was predominant in the 2015 rotavirus season, indicating that they were exposed to a natural infection. In-vitro studies have shown that high titres of neutralising activity in breastmilk resulted in a reduction of rotavirus vaccine virus titres.^30^ Theoretically, rotavirus antibodies could neutralise virus vaccine if breastmilk was in the stomach of the infant at the time of vaccination, which could decrease the titre of vaccine virus reaching the gut. Breastfeeding could thus potentially have influenced the shedding of Rotarix in our study. We did not have an oral vaccine comparator because the study was primarily designed to measure safety and immunogenicity compared with placebo, with a view to further development of this vaccine platform. Future studies to compare shedding after vaccination between parenteral and a live oral vaccines might prove useful in exploring a reduction in shedding as an alternative measure of field efficacy.

The results of this first clinical trial of the P2-VP8-P[8] subunit rotavirus vaccine in toddlers and infants support further development and testing of the platform. The P2-VP8-P[8] vaccine was found to be safe and immunogenic, with evidence that it might provide protection against rotavirus disease in infants. Vaccination with P2-VP8-P[8] did not adversely affect the subsequent immune response to Rotarix, which has implications for the concomitant use of parenteral and oral vaccines to achieve optimal protection against severe rotavirus disease. Since live oral and inactivated rotavirus vaccines are highly likely to confer protection by distinct mechanisms, concurrent vaccination could potentially provide optimal protection in the field. Demonstration of reduced rotavirus shedding following P2-VP8-P[8] vaccination raises the possibility of using this model as a surrogate marker of field efficacy, although further studies are needed to explore this potential.
